# Teledentistry: A Future Solution in the Diagnosis of Oral Lesions: Diagnostic Meta-Analysis and Systematic Review

**DOI:** 10.1089/tmj.2022.0426

**Published:** 2023-11-10

**Authors:** Eszter Uhrin, Zsuzsanna Domokos, László Márk Czumbel, Tamás Kói, Péter Hegyi, Péter Hermann, Judit Borbély, Bianca Golzio Navarro Cavalcante, Orsolya Németh

**Affiliations:** ^1^ Department of Community Dentistry, Semmelweis University, Budapest, Hungary.; ^2^ Centre for Translational Medicine, Semmelweis University, Budapest, Hungary.; ^3^ Department of Periodontology, Semmelweis University, Budapest, Hungary.; ^4^ Department of Stochastics, Institute of Mathematics, Budapest University of Technology and Economics, Budapest, Hungary.; ^5^ Institute for Translational Medicine, Szentágothai Research Centre, Medical School, University of Pécs, Pécs, Hungary.; ^6^ Division of Pancreatic Diseases, Heart and Vascular Center, Semmelweis University, Budapest, Hungary.; ^7^ Department of Prosthodontics and Semmelweis University, Budapest, Hungary.; ^8^ Department of Oral Biology, Semmelweis University, Budapest, Hungary.

**Keywords:** telediagnosis, e-health, oral medicine, oral cancer, precancerous lesion

## Abstract

**Introduction:**

Teledentistry (TD) can offer a wide range of possibilities in the field of oral medicine. Oral potentially malignant disorders (OPMDs) are hard to detect, and even harder to diagnose correctly. With the help of TD, OPMDs can be detected and diagnosed by a remote specialist. Our aim was to investigate whether TD could provide a reliable diagnostic method compared with clinical oral examination (COE) in the diagnosis of OPMDs.

**Methods:**

A systematic search was conducted in three databases (Medline, EMBASE, CENTRAL) until November 2021. We included studies that compared telediagnosis and COE, both made by experts. Pooled specificity and sensitivity were calculated and visualized on a two-dimensional plot. Risk of bias was assessed using the QUADAS-2 tool, and the level of evidence is shown with the Grades of Recommendation, Assessment, Development and Evaluation (GRADE) tool.

**Results:**

Of the 7,608 studies, 13 were included in the qualitative and 9 in the quantitative synthesis. Using TD tools in the detection of oral lesions (OLs) showed high specificity (0.92 confidence interval [95% CI] = 0.59–0.99) and sensitivity (0.93 95% CI = 0.17–1.00). In the differential diagnosis of lesions, we found high sensitivity and specificity (0.942 95% CI = 0.826–0.982 and 0.982 95% CI = 0.913–0.997), respectively. We summarized the available data on time-effectiveness, screening person, referral decision, and technical settings.

**Conclusion:**

Detecting OLs with TD tools might lead to earlier diagnosis, treatment, and stricter follow-up of OPMD. TD may offer a great substitution for COE in the diagnosis of OLs, and thus, fewer referrals could be made to special care, resulting in a greater number of treated OPMDs.

## Introduction

Teledentistry (TD), as part of telemedicine, is an information technology-based diagnosis, treatment, and education delivery system in the dental field.^1^ The recent rise of the use of TD due to COVID-19 has shown its potential for implementing different solutions to primary dental care.^2^

TD involves store-and-forward (SAF) solutions, which allow the patient–doctor communication to take part both at different time points (e.g., via a chat application) and in real-time (RT) solutions that require both the patient and the doctor to be simultaneously present in the online space (e.g., videoconference). Through these solutions, the transfer of electronic health records, teleconsultation, telediagnosis, online therapy planning, follow-up, remote patient monitoring, online research, and tele-education can be applied to dentistry.^1^

As visual information can include important details about a clinical case, a photograph-based telediagnosis depends on image resolution.^3^ Teledermatology, where evidence for telediagnosis is based on image capturing, is one of the most investigated and developing fields in telemedicine.^4^ Recent research in teledermatology shows contradictory findings about the diagnostic accuracy of the field,^5,6^ however, this research has not yet been performed in oral medicine (OM).

The correct diagnosis of premalignant lesions is essential. Due to the fact that oral cancer (OC) in an early stage is asymptomatic, it lacks patient-reported symptoms: ulceration, bleeding, and induration.^7^ In developing countries such as India and Malaysia where smokeless tobacco (e.g., betel chewing) is popular, the problem arises.^8^ Other risk factors can include infections, solar radiation, dental hygiene, and genetic factors.^8^ A low-resource setting and the lack of specialists in rural areas lengthen the diagnosing procedure, allowing the manifestation of oral potentially malignant disorders (OPMDs). TD could help in bridging this problem. Providing high-risk populations with a general oral mucosa screening could decrease the rising amount of OC.

Diagnosing oral lesions (OL) can be difficult due to the variability of presence and the lack of training in OM.^9^ Detection and follow-up of OPMDs at an early stage could stop their manifestation to OC. Also, the similarities between precancerous lesions and some benign lesions cause difficulties in differential diagnosis.^7^ Therefore, many unnecessary referrals are made by primary dentists to OM specialists, resulting in wait time and travel difficulties for patients.^10^

TD has the potential to connect patients with dentists via the online space, thus improving access to care in rural areas.^11^

This systematic review and meta-analysis aimed to collect available data on how OM could benefit from TD solutions. Due to the lack of data about different TD tools and concise data about the use of TD in OM, an extended summary is needed.

Our aim is to investigate the existing TD tools in the diagnosis of OLs using clinical oral examination (COE) as a reference standard. The main outcome is the presence and the differential diagnostic accuracy of OPMDs, which can lead to lowering the number of referrals to special care.

## Methods

Reporting of this systematic review and meta-analysis is based on the recommendation of the PRISMA 2020 guideline,^12^ while we followed the Cochrane Handbook.^12^ The protocol of the study was registered on PROSPERO (registration number CRD 42021282645) with no deviations from it.

### SEARCH STRATEGY AND ELIGIBILITY CRITERIA

The systematic search was conducted on October 25, 2021, and was revised on October 11, 2022, in the following electronic databases: MEDLINE (via PubMed), Embase, and the Cochrane Central Register of Controlled Trials (CENTRAL). The search key we used in each database is detailed in Supplementary Data S1.

The search applied no filters. Only English language articles were considered eligible. In addition, the reference lists of eligible studies were also searched. Nonoriginal articles (reviews, editorials, letters, and comments) and nonpeer-reviewed articles (gray literature) were excluded. *In vitro* and animal studies were also excluded.

Study eligibility was determined based on the PICO framework: adults with suspected OLs (P), who were examined using TD tools (that included imaging, I) were acceptable with a reference standard (C) of COE or biopsy. Specificity and sensitivity, positive predictive value (PPV), and negative predictive value (NPV) were considered the included outcomes (O). Regarding the study type, observational studies were considered eligible.

### SELECTION PROCESS

The selection was performed by two independent review authors (E.U. and Z.D.). After the duplicates were removed using reference management software (EndNote X9, Clarivate Analytics), the two authors independently screened titles, abstracts, and then full texts. Disagreements were resolved by a third independent reviewer (O.N.).

### DATA COLLECTION PROCESS

Data were collected by two authors independently (E.U. and Z.D.) based on a predefined data collection form. The disagreements were resolved by a third independent author (O.N.).

The following data were extracted: first author, year of publication, type of study, country, study population, mean age, surveyed risk factors, number of participants, target condition definition, person conducting telediagnosis, type of photography tool, description of TD tool, and reference standard.

### DIAGNOSTIC ACCURACY MEASURES

The comparison of a new diagnostic test (TD) with the gold standard of the field (COE) is conventionally summarized in a two-by-two table called the diagnostic contingency table. It contains the numbers of true-positive (TP), false-positive (FP), true-negative (TN), and false-negative (FN) cases. The most common measures calculated based on the table include sensitivity, specificity, accuracy, and PPV and NPV. All these data were collected in a predefined data table.

### ORAL PREMALIGNANT OR MALIGNANT DISEASE

We established a detailed diagnosis classification table based on Haron's predefined list^13^ to define OPMD lesions in those articles that have only the final diagnosis listed. We considered the WHO classification of OLs^14^ as the standard decision base in cases of questionable diagnosis. The final table was discussed with an OM expert (O.N.).

### RISK-OF-BIAS ASSESSMENT

Based on the Cochrane Handbook for Systematic Reviews of Diagnostic Test Accuracy,^12^ we used the QUADAS-2 tool^15^ for risk-of-bias assessment. This was performed by two review authors (E.U. and Z.D.). Disagreements were resolved by a third independent author (O.N.).

### CERTAINTY OF EVIDENCE

Certainty of evidence was evaluated based on the Grades of Recommendation, Assessment, Development and Evaluation (GRADE) workgroup's recommendations.^16^ The endpoints of the outcomes were assessed by two independent reviewers (E.U. and Z.D.). In case of disagreement, a third independent reviewer resolved it (O.N.).

We made the GRADE evidence profiles with GRADEpro GDT Software^17^ for the investigated outcomes.

### SYNTHESIS METHODS

Two by two contingency tables were extracted from the studies containing TP, FP, FN, and TN values. Where studies published data corresponding to more than one remote expert, we randomly selected one of them. We repeated the analysis using the contingency tables of previously nonselected experts as a sensitivity analysis.

We used the bivariate model of Chu and Cole and Reitsma et al.^18,19^ Ellipsoids of this analysis reflect the weights of the studies according to the method by Burke et al.^20^ Univariate analyses of sensitivity and specificity were performed using the generalized mixed-effect approach.^21^ Statistical analyses were carried out using the online tool described in Freeman et al.^22^ and the meta package of R statistical software (version 4.1.2.). The statistical analyses followed the advice of Harrer et al.^23^ Further details of the synthesis methods are described in Supplementary Data S2.

## Results

### SEARCH AND SELECTION

**Fig. 1.**
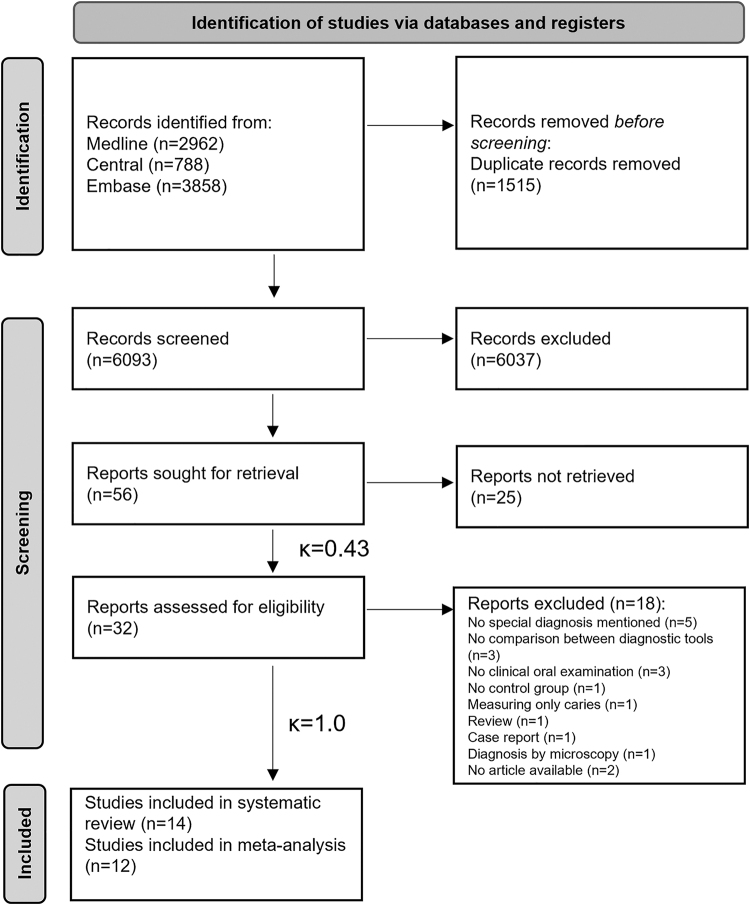
PRISMA flowchart.

In all, 7,608 studies were identified by our systematic search, 13 of which were included in the qualitative synthesis.^3,10,13,24–33^ By the end of the process, 10 articles were found eligible for the quantitative synthesis,^3,10,13,25,26,29–32,34^ and 4 articles were excluded due to lack of data.^24,27,28,33^ The selection process and Cohen's kappa values are presented using a PRISMA flowchart (*Fig. 1*).

### BASIC CHARACTERISTICS OF INCLUDED STUDIES

The baseline characteristics of the studies included in the qualitative analysis are detailed in *Table 1*.

**Table 1.** Characteristics of the Studies Included in Qualitative Analysis

AUTHORS (YEAR)	STUDY SITE	STUDY DESIGN	N^O^ OF PARTICIPANTS	MEAN AGE (YEARS)	INCLUSION CRITERIA	EXCLUSION CRITERIA	RISK FACTOR SURVEYED	TARGET CONDITION DEFINITION	INDEX TEST			REFERENCE STANDARD
									PERSON CONDUCTING REMOTE DIAGNOSIS	TYPE OF PHOTOGRAPHY TOOL	DESCRIPTION OF TELEDENTISTRY TOOL	
Birur^a^ et al. (2015)^25^	India	Cohort	3,440	18–85	Targeted group from two rural villages, opportunistic group from oral surgeon's screening	N/A	Smoking tobacco, chewing betel leaf/gutka, regular use of alcohol	N/A	Not applicable	Mobile phone	Sana application was designed that is integrated with OpenMRS (medical record system)	Not applicable
Birur et al. (2019)^24^	India	Cross-sectional	3,445	18–57	Workers of a pipeline factory	N/A	High risk of tobacco use	WHO	Remote specialist using m-health	Mobile phone	Mobile phone-based questionnaire and photographs of oral cavity	Screening by onsite specialist
Flores et al. (2022)^34^	Brazil	Cross-sectional	100	51.3	Patients referred to one of the three included oral medicine centers	N/A	N/A	Exact lesion diagnosis	Remote dentist	Mobile phone (at least 10 MP resolution) or camera	Mobile phone application that includes patient data, and characteristics of the lesion and photographs of the lesion	Expert professor
Gomes and colleagues (2017)^26^	Brazil	Prospective	55	60–80+	>40 years old, smoking	N/A	Smoking, alcohol consumption, oral/pharyngeal cancer in family	Grouped lesions (9)	2 trained examiners (>3 years of experience)	Mobile phone	Newly developed app: videos of oral cavity and in-app data	Examiners with experience in oral diagnosis
Fonseca^a^ et al. (2021)^27^	Brazil	Cross-sectional	113	52	>5 years of age with oral lesion	Difficulties with mouth opening	N/A	Final diagnosis, listed	Oral medicine and oral pathology professors (15 years of experience)	iPhone 5, 8 MP camera	Photographs were e-mailed with clinical implication to evaluators	COE or biopsy
Haron et al. (2017)^3^	Malaysia	Cross-sectional	8	N/A	8 targeted lesions, 8 normal or variant of normal	Not applicable	Not applicable	OPMD/non-OPMD/normal	Oral medicine specialist	5–13 MP mobile phone camera	Photographs taken by mobile phone, retrieved later for review	COE by oral medicine specialist
Haron et al. (2020)^13^	Malaysia	Prospective	355	53.9	Adults >18 years old referred by primary dentist	N/A	Alcohol consumption, smoking, betel-chewing	Presence of lesion/normal variant, predetermined table	Off-site specialist	13 MP mobile phone	Using MeMoSa application	COE by oral medicine or oral surgery specialist
Namakian^a^ et al. (2012)^28^	United States	Observational	29	47	Patients of Special Care Clinic: intellectual disability, cerebral palsy, Down syndrome, autism, seizures, HIV, liver disease, neurologic disorders, stroke, schizophrenia	If patient was not able to cooperate for record collection (at least intraoral and extraoral photographs)	N/A	Community-based treatment or referral to dentist	Study dentists	Intraoral camera, point-and-shoot camera	COE followed by an evaluation questionnaire, after 3-week-washout period a virtual examination was conducted	Study dentists
Perdoncini et al. (2021)^29^	Brazil	Cross-sectional	33	53	>18-year-old patients referred to the clinic with oral lesion	Symptoms but no oral sign	N/A	Diagnosis per lesion	Oral medicine specialist	iPhone SE	The dentist sent the photographs to specialist via WhatsApp, and a video call was initialized	Oral medicine specialist
Petruzzi and De Benedittis (2016)^10^	Italy	Cross-sectional	96	N/A	Referred patients by general dentists/oral hygienist/physician OR patient	N/A	N/A	Traumatic, infective, preneoplastic/neoplastic, autoimmune, not diagnosable	Oral medicine specialist	Mobile phone	Photographs sent via WhatsApp, and after clinical examination was made. Biopsy was made where needed.	Oral medicine specialist
Tesfalul et al. (2016)^30^	United States	Observational	23	45.5	Referred adult patients with complicated oral lesion, receiving care via the mobile oral telemedicine system	<18 years, incomplete telemedicine consultation, failure to obtain consent	N/A	Cancer, infection, fracture, benign mass, dermatologic conditions, other	Specialist	HTC myTouch mobile phone	Mobile telemedicine application including image-sharing and clinical data	COE conducted by dentist
Torres-Pereira et al. (2008)^31^	Brazil	Cross-sectional	25	N/A	People with oral lesion	People without oral lesion	N/A	Predefined list of terms	Oral medicine specialist (10 years of experience)	FUJI S7000 digital camera	Images sent via e-mail, with an electronic form containing patient's data	COE by oral medicine specialist or biopsy when needed
Torres-Pereira et al. (2013)^32^	Brazil	Cross-sectional	60	N/A	People with oral lesion	N/A	N/A	Predefined list of terms	Oral medicine specialist	Canon EOS 300 Rebel digital camera	Images sent via e-mail, with an electronic form containing patient's data	Biopsy
Vinayagamoorthy^a^ et al. (2019)^33^	India	Cross-sectional	131	37.34	Patients included from oral screening programs	Having problem with comprehension, limited mouth opening	Medical and habit history	Normal/abnormal, exact lesion diagnosis	Trained and calibrated examiner	Samsung mobile phone	A set of five photographs were made, then sent to specialist via WhatsApp	Trained and calibrated examiner

^a^ Study included only in systematic review.

COE, clinical oral examination; OPMDs, oral potentially malignant disorders.

### PRIMARY OUTCOMES

#### Oral lesion detection with TD tools

**Fig. 2.**
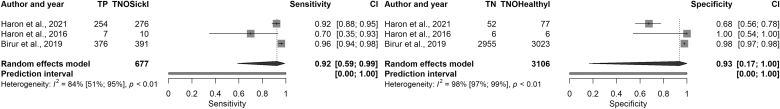
Sensitivity and specificity of diagnosing the presence of oral lesions with teledentistry tools. CI, confidence interval; TP, true positive; TN, true negative.

Our pooled analysis of 3 articles^3,13,24^ including 3,783 patients showed that TD tools can be a reliable option for replacing face-to-face dental visits in the detection of OLs (sensitivity: 0.92 confidence interval [CI] = 0.59–0.99; specificity: 0.93 CI = 0.17–1.00) (*Fig. 2*). The data used for this analysis are shown in *Supplementary Table S1*. The examined outcome showed a substantially heterogenous population (sensitivity: *I*^2^ = 84%, *p* < 0.01; specificity: *I*^2^ = 98%, *p* < 0.01). Fitting the bivariate model was not possible in this analysis due to the small number of available studies.

#### Diagnosing oral premalignant lesions or oral cancer

As the exact number of TP, TN, FP, and FN lesions was not mentioned in every article, the abovementioned classification table was able to help us resolve the problem, as can be seen in *Supplementary Table S2*.

**Fig. 3.**
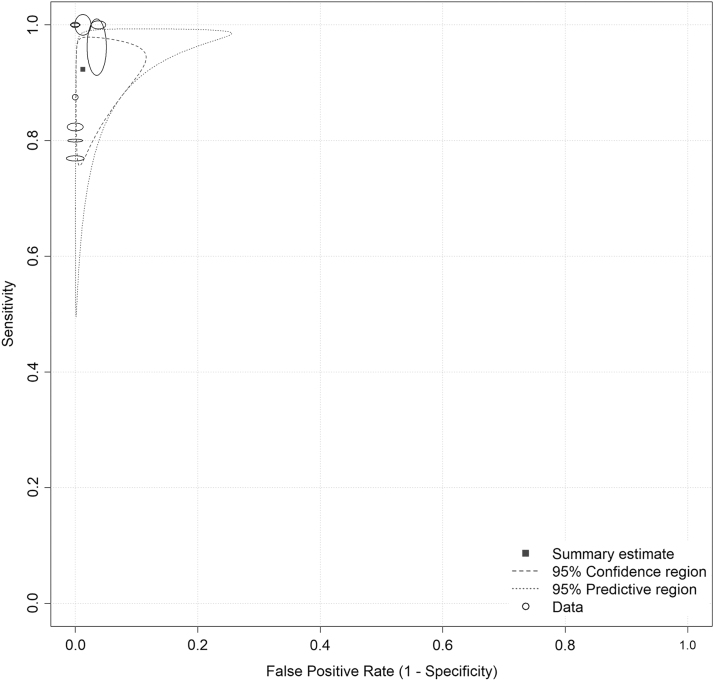
Sensitivity and specificity of diagnosing the oral premalignant/malignant lesions with teledentistry tools-2D analysis.

We assessed the available data (*Supplementary Table S3*) for the diagnosis of OPMD or OC lesions. Estimated high sensitivity and specificity of nine pooled articles^3,10,13,26,29–32,34^ showed that TD can be a reliable tool for differential diagnosis of OLs (sensitivity: 0.923 CI = 0.835–0.966; specificity: 0.987 CI = 0.947–0.997), as shown in *Figure 3* and *Supplementary Figure S1*. Heterogeneity is low (the size of the prediction region is moderate; sensitivity: *I*^2^ = 0%, *p* = 0.66; specificity: *I*^2^ = 0%, *p* = 1.00), as shown in *Supplementary Figure S1*.

Conducting sensitivity analysis, the resulting sensitivity and specificity are 0.953 (CI = 0.856–0.986) and 0.983 (CI = 0.930–0.996), respectively. See Supplementary Data for further details of the extractions (*Supplementary Table S4*). This result confirmed the findings of the main analysis; thus, the outcome shows that the results do not depend on the examiner's knowledge (*Supplementary Figs. S2 and S3*).

### SECONDARY OUTCOMES

#### Time-effectiveness

No statistical analysis could be performed on time-effectiveness due to the heterogeneity of reported data. Namakian et al.^28^ measured the difference in time during in-person examination (mean: 4.2 min, SD: 1.6) and virtual examination (2.83 min, SD: 1.0). Perdoncini et al.^29^ listed the transportation time (mean: 58.3 min, SD: 52.9), which shows that mostly people from rural areas attended the clinic. The COE took 10.44 min on average (SD: 2.88). In the case of the WhatsApp messaging platform, Petruzzi and De Benedittis^10^ measured an average distance of 70 km (SD: 78 km).

#### Screening person

Birur et al.^25^ showed that a trained frontline health care worker could detect OPMD with a PPV of 45%. They also studied the efficacy of community health care workers (CHWs) in the diagnosis of the presence of OL, where they found a kappa score of 0.92 between CHWs and onsite specialists.^24^ Therefore, not only dentists but also trained CHWs could take part in the detection of OLs.

#### Referral decision

The referral decision was measured in four articles,^3,13,27,28^ however, its policy showed variability. Fonseca et al.^27^ recorded that an average of 35.4% of the examined patients could be treated in primary dental care. Haron et al.^13^ recorded a kappa value of 0.892 (CI: 0.843–0.940) for referrals using a predefined list.

#### Technical setting

The technical setting of TD tools varied in the available articles. Haron et al.^3^ found a correlation between using better resolution mobile phones as photograph-taking devices in diagnosing lesion category: the higher the resolution, the higher the diagnostic agreement (accuracy: 0.515–0.881). Using a better mobile phone helped not only in better detection (sensitivity: 0.813), but also in correct diagnosis (sensitivity: 0.93).

Perdoncini et al.^29^ investigated the accuracy of RT video consultation with a specialist; 91% of the photographs were of good quality, and the internet connection was stable in 58% of the cases.

A professional camera was used in two articles^31,32^ to simulate primary dental care settings. The images then were sent to the specialist via e-mail, thus avoiding internet connection problems.

Receiving photographs and testing the role of free chat applications in dentistry can simulate population-based screening.^10,29,33^ Not only dentists, but also dental hygienists and patients sent homemade photographs of OLs. WhatsApp was tested by Vinayagamoorthy et al.,^33^ where a sensitivity of 0.981 (examiner 1) and 0.987 (examiner 2) was found for exact lesion diagnosis.

### RISK-OF-BIAS ASSESSMENT

Using the QUADAS-2 tool,^15^ our results for risk-of-bias assessment are presented in *Supplementary Figures S4*, *S5*, and *S6*. Most of the domains showed low risk of bias, however, having inappropriate exclusion criteria in patient selection in one article meant that only people with risk factors were involved in the actual study,^27^ and in another study, the patients were selected by having/not having OL/OPMD/OC.^3^ The index test result domain has unclear risk in those articles where COE (index test) was made by the same person who made the telediagnosis with a fixed, predefined washout period.^27,28,30^

Due to the small number of available studies, publication bias could not be assessed by performing the methodology of Deeks et al.^35^ Certainity of evidence Table can be seen in *Supplementary Table S5*. The PRISMA checklist can be seen in *Supplementary Table S6*.

## Discussion

According to the high sensitivity and specificity in our study, TD could successfully help in primary dental care in the field of OM. Store-and-forward systems could be used in telediagnosis (e.g., photographs sent via e-mail), and RT solutions could be used in teleconsultation (e.g., video consultation).^29^

Visual examination is extremely important (and remains the primary diagnostic tool) in both dermatology and OM, causing a rapid technical rise in both teledermatology and TD.^36^ Image-based diagnosis by a specialist may help primary dental care facilities in the decision of referral to special care.

Also, TD could promote organizing population-based OL screening programs in the future.^25^ The differential diagnosis of OLs is important due to treatment planning reasons. TD could support general dentists in OL management through connection with OM specialists, enabling early diagnosis and correct treatment of oral (pre)malignant lesions.^34,36,37^ Knowing the best available treatment and prevention could lead to less manifestation of OC. Furthermore, referring only OPMDs and malignant OLs might result in a higher percentage of treated patients.

As the prevalence of OC is rising, the correct diagnosis of premalignant lesions is essential. In developing countries, low-resource settings and the lack of specialists in rural areas make the diagnosing procedure longer, allowing manifestations of OPMD. TD could help in solving this problem.

TD tools in the diagnosis of OLs show high accuracy, however, there are weaknesses in the available data that need to be mentioned. The protocol of diagnosis by the same person was followed by several articles, which despite the washout time (average 3 weeks) still brings into question the reliability and correctness of the results.^3,13,28,33^ Also, in one case, the general dentist could send his or her own diagnostic opinion, compromising the impartiality of the diagnosis.^30^

Education in the field of OM starts during the predoctoral phase, however, it varies afterward. A huge difference can be spotted regarding specialists: for example, in the United States, this field is a recognized dental specialty of the American Dental Association,^38^ however, in Europe, it varies from country to country, and a consensus has not been made yet about the competencies and limits of OM specialists.^39^ This fact questions the use of the term OM “specialist” in the articles. In addition, experience in the field does not guarantee a correct diagnosis using TD tools. Lack of information in the online form and the lack of in-person personalized questions may complicate the diagnosing process.

Referring every patient with an OL to special care might overload OM praxis. A high number of referrals may be avoidable using a pretriage system^40^ or population-based screening.^24^ This could increase the efficacy of patient flow in OM practices.

### OL DETECTION

OC is unique compared with other cancers because visual detection at an early stage is possible with COE,^41^ however, we cannot predict the level of dysplasia based on clinical images. Diagnosing the presence and type of OL could lead to targeted treatment and prevention of manifestation to OC.^41^ Our results showed both high specificity and sensitivity in the detection of OLs, meaning that TD tools could be used as a pretriage system in the diagnosis of OLs.

### DIFFERENTIAL DIAGNOSIS OF OLs

Correct diagnosis of an OL is necessary before treatment, however, not all patients need to be referred to an OM specialist. Our study showed high sensitivity, meaning that TP OPMD could be successfully diagnosed via TD image-sharing tools (e-mail, free chat applications, cloud-based storage applications, etc.). We found high specificity, meaning that TN lesions that do not necessitate referral to a specialist are correctly diagnosed, affirming previous articles' clinical data.^3,13^

### STRENGTHS

According to our knowledge, this is the first meta-analysis on this specific topic. We used rigorous methodology and followed a previously registered protocol. We selected for eligibility only those articles in which the clinical diagnosis (reference standard) was made only by doctors. Due to the strict inclusion criteria, we gained higher data quality (e.g., we excluded articles that did not include a reference standard). We did not include publications with retrospective or case–control designs.

### LIMITATIONS

Regarding the limitations of this article, within the available data regarding the detection of OLs, we found significant heterogeneity among the included articles. The reason for this, despite the strict inclusion criteria, could be the included heterogenous population (e.g., a preselected population or factory workers). The methodology of applied TD tools was heterogeneous, meaning that RT and SAF systems were pooled together. The referral decision was not defined in each of our selected articles, and so, we could not include it in a quantitative analysis.

### IMPLICATION FOR PRACTICE

A future photograph-taking protocol for OLs could be used in primary dental care. We suggest establishing the technical setting first, as telecommunication tools require a firm technical background. Also, a standardized questionnaire could be used in the future to provide more details about the patient.

### IMPLICATION FOR RESEARCH

We suggest that researchers use biopsy as a reference standard (replacing COE) to have an objective diagnosis. Furthermore, exact lesion diagnosis could be helpful in comparison with other publications' results, and referral decisions could be counted based on these data.

## Conclusions

Detecting OLs with TD tools could lead to early diagnosis, treatment, and stricter follow-up of OPMDs. TD offers a great substitute for face-to-face dental visits in the detection and differential diagnosis of OLs, and thus, fewer referrals could be made to special OM care. Being a time-effective solution, when conducted in a proper way, access to care could be widened with the use of these tools.

## Supplementary Material

Supplemental data

Supplemental data

Supplemental data

Supplemental data

Supplemental data

Supplemental data

Supplemental data

Supplemental data

Supplemental data

Supplemental data

Supplemental data

Supplemental data

Supplemental data

Supplemental data

## References

[1] Khan SA, Omar H. Teledentistry in practice: Literature review. Telemed J E Health 2013;19(7):565–567; doi: 10.1089/tmj.2012.0200 23672799

[2] Plaza-Ruíz SP, Barbosa-Liz DM, Agudelo-Suárez AA. Impact of COVID-19 on the knowledge and attitudes of dentists toward teledentistry. JDR Clin Trans Res 2021;6(3):268–278; doi: 10.1177/2380084421998632 33632011

[3] Haron N, Zain RB, Nabillah WM, et al. Mobile phone imaging in low resource settings for early detection of oral cancer and concordance with clinical oral examination. Telemed J E Health 2017;23(3):192–199; doi: 10.1089/tmj.2016.0128 27541205

[4] Wang RH, Barbieri JS, Kovarik CL, et al. Synchronous and asynchronous teledermatology: A narrative review of strengths and limitations. J Telemed Telecare 2022;28(7):533–538; doi: 10.1177/1357633X221074504 35108130

[5] Lee JJ, English JC, 3rd. Teledermatology: A review and update. Am J Clin Dermatol 2018;19(2):253–260; doi: 10.1007/s40257-017-0317-6 28871562

[6] Andrees V, Klein TM, Augustin M, et al. Live interactive teledermatology compared to in-person care—A systematic review. J Eur Acad Dermatol Venereol 2020;34(4):733–745; doi: 10.1111/jdv.16070 31715035

[7] Silverman S Jr. Early diagnosis of oral cancer. Cancer 1988;62(8 Suppl):1796–1799; doi: 10.1002/1097-0142(19881015)62:1+<1796::aid-cncr2820621319>3.0.co;2-e 3167796

[8] Kumar M, Nanavati R, Modi TG, et al. Oral cancer: Etiology and risk factors: A review. J Cancer Res Ther 2016;12(2):458–463; doi: 10.4103/0973-1482.186696 27461593

[9] Ergun S, Ozel S, Koray M, et al. Dentists' knowledge and opinions about oral mucosal lesions. Int J Oral Maxillofac Surg 2009;38(12):1283–1288; doi: 10.1016/j.ijom.2009.07.004 19651489

[10] Petruzzi M, De Benedittis M. WhatsApp: A telemedicine platform for facilitating remote oral medicine consultation and improving clinical examinations. Oral Surg Oral Med Oral Pathol Oral Radiol 2016;121(3):248–254; doi: 10.1016/j.oooo.2015.11.005 26868466

[11] Estai M, Kanagasingam Y, Tennant M, et al. A systematic review of the research evidence for the benefits of teledentistry. J Telemed Telecare 2018;24(3):147–156; doi: 10.1177/1357633X16689433 28118778

[12] Higgins JPT, Thomas J, Chandler J, et al. Cochrane Handbook for Systematic Reviews of Interventions version 6.3 2022. Cochrane, Available from: www.training.cochrane.org/handbook [Last accessed: February, 2022].

[13] Haron N, Zain RB, Ramanathan A, et al. m-Health for early detection of oral cancer in low- and middle-income countries. Telemed J E Health 2020;26(3):278–285; doi: 10.1089/tmj.2018.0285 31081720

[14] Ramadas K, Lucas E, Thomas G, et al. A digital manual for the early diagnosis of oral neoplasia. Available from: https://screening.iarc.fr/atlasoral.php [Last accessed: August 6, 2022].

[15] Whiting PF, Rutjes AW, Westwood ME, et al. QUADAS-2: A revised tool for the quality assessment of diagnostic accuracy studies. Ann Intern Med 2011;155(8):529–536; doi: 10.7326/0003-4819-155-8-201110180-00009 22007046

[16] Schünemann HBJ, Guyatt G, Oxman A, et al. GRADE handbook for grading quality of evidence and strength of recommendations. Updated October 2013. The GRADE Working Group, 2013. Available from: guidelinedevelopment.org/handbook. 2013.

[17] GRADEpro GDT: GRADEpro Guideline Development Tool [Software]. McMaster University and Evidence Prime Afgo. 2021. Available from: https://www.gradepro.org/.

[18] Chu H, Cole SR. Bivariate meta-analysis of sensitivity and specificity with sparse data: A generalized linear mixed model approach. J Clin Epidemiol 2006;59(12):1331–1332; doi: 10.1016/j.jclinepi.2006.06.011 17098577

[19] Reitsma JB, Glas AS, Rutjes AWS, et al. Bivariate analysis of sensitivity and specificity produces informative summary measures in diagnostic reviews. J Clin Epidemiol 2005;58(10):982–990; doi: 10.1016/j.jclinepi.2005.02.022 16168343

[20] Burke DL, Ensor J, Snell KIE, et al. Guidance for deriving and presenting percentage study weights in meta-analysis of test accuracy studies. Res Synth Methods 2018;9(2):163–178; doi: 10.1002/jrsm.1283 29115060

[21] Stijnen T, Hamza TH, Özdemir P. Random effects meta-analysis of event outcome in the framework of the generalized linear mixed model with applications in sparse data. Stat Med 2010;29(29):3046–3067; doi: 10.1002/sim.4040 20827667

[22] Freeman SC, Kerby CR, Patel A, et al. Development of an interactive web-based tool to conduct and interrogate meta-analysis of diagnostic test accuracy studies: MetaDTA. BMC Med Res Methodol 2019;19(1):81; doi: 10.1186/s12874-019-0724-x 30999861 PMC6471890

[23] Harrer M, Cuijpers P, Furukawa TA, et al. Doing Meta-Analysis with R A Hands-on Guide. CRC Press, Taylor & Francis Group; 2022.

[24] Birur N, Gurushanth K, Patrick S, et al. Role of community health worker in a mobile health program for early detection of oral cancer. Indian J Cancer 2019;56(2):107–113; doi: 10.4103/ijc.IJC_232_18 31062727

[25] Birur PN, Sunny SP, Jena S, et al. Mobile health application for remote oral cancer surveillance. J Am Dent Assoc 2015;146(12):886–894; doi: 10.1016/j.adaj.2015.05.020 26610833

[26] Bonan P, Gomes MS, Bonan PRF, et al. Development of a mobile application for oral cancer screening. Technol Health Care 2017;25(2):187–195; doi: 10.3233/THC-161259 27689559

[27] Fonseca BB, Perdoncini NN, da Silva VC, et al. Telediagnosis of oral lesions using smartphone photography. Oral Dis 2021; doi: 10.1111/odi.13972 10.1111/odi.13972 34289201

[28] Namakian M, Subar P, Glassman P, et al. In-person versus “virtual” dental examination: Congruence between decision-making modalities. J Calif Dent Assoc 2012;40(7):587–595. 22916380

[29] Perdoncini NN, Schussel JL, Amenábar JM, et al. Use of smartphone video calls in the diagnosis of oral lesions: Teleconsultations between a specialist and patients assisted by a general dentist. J Am Dental Assoc 2021;152(2):127–135; doi: 10.1016/j.adaj.2020.10.013 33494867

[30] Tesfalul M, Littman-Quinn R, Antwi C, et al. Evaluating the potential impact of a mobile telemedicine system on coordination of specialty care for patients with complicated oral lesions in Botswana. J Am Med Inform Assoc 2016;23(e1):e142–e145; doi: 10.1093/jamia/ocv140 26510877 PMC4954624

[31] Torres-Pereira C, Possebon RS, Simoes A, et al. Email for distance diagnosis of oral diseases: A preliminary study of teledentistry. J Telemed Telecare 2008;14(8):435–438; doi: 10.1258/jtt.2008.080510 19047454

[32] Torres-Pereira CC, Morosini IA, Possebon RS, et al. Teledentistry: Distant diagnosis of oral disease using e-mails. Telemed J E Health 2013;19(2):117–121; doi: 10.1089/tmj.2012.0087 23356381 PMC3576903

[33] Vinayagamoorthy K, Acharya S, Kumar M, et al. Efficacy of a remote screening model for oral potentially malignant disorders using a free messaging application: A diagnostic test for accuracy study. Aust J Rural Health 2019;27(2):170–176; doi: 10.1111/ajr.12496 30942518

[34] Flores APdC, Roxo-Gonçalves M, Batista NVR, et al. Diagnostic accuracy of a telediagnosis service of oral mucosal diseases: A multicentric survey. Oral Surg Oral Med Oral Pathol Oral Radiol 2022;134(1):65–72; doi: 10.1016/j.oooo.2022.02.005 35422409

[35] Deeks JJ, Macaskill P, Irwig L. The performance of tests of publication bias and other sample size effects in systematic reviews of diagnostic test accuracy was assessed. J Clin Epidemiol 2005;58(9):882–893; doi: 10.1016/j.jclinepi.2005.01.016 16085191

[36] Irving M, Stewart R, Spallek H, et al. Using teledentistry in clinical practice as an enabler to improve access to clinical care: A qualitative systematic review. J Telemed Telecare 2018;24(3):129–146; doi: 10.1177/1357633X16686776 28092220

[37] Carrard VC, Roxo Goncalves M, Rodriguez Strey J, et al. Telediagnosis of oral lesions in primary care: The EstomatoNet Program. Oral Dis 2018;24(6):1012–1019; doi: 10.1111/odi.12851 29505701

[38] American Dental Association's recognized specialities. Available from: https://ncrdscb.ada.org/en/dental-specialties/specialty-definitions [Last accessed: October 5, 2022].

[39] Bez C, Sklavounou A, Carrozzo M. Oral medicine in Europe: Past, present and future. Br Dent J 2017;223(9):726–728; doi: 10.1038/sj.bdj.2017.891 29074939

[40] Estai M, Kanagasingam Y, Xiao D, et al. A proof-of-concept evaluation of a cloud-based store-and-forward telemedicine app for screening for oral diseases. J Telemed Telecare 2016;22(6):319–325; doi: 10.1177/1357633X15604554 26377126

[41] Mehrotra R, Gupta DK. Exciting new advances in oral cancer diagnosis: Avenues to early detection. Head Neck Oncol 2011;3:33; doi: 10.1186/1758-3284-3-33 21798030 PMC3170277

