# Supplementary material for: Teledentistry: A Future Solution in the Diagnosis of Oral Lesions: Diagnostic Meta-Analysis and Systematic Review
Source: Telemed J E Health. 2023 Nov 10;29(11):1591–600. doi: 10.1089/tmj.2022.0426 (PMC10654653; doi:10.1089/tmj.2022.0426)
Supplement: Supplemental data [file Suppl_DataS1.docx]

**Supplementary Material 1.** Search query

(telemedicine OR teledentistry OR telehealth OR telediagnosis OR telecons* OR ehealth OR smartphone OR phone OR remote OR app) AND (oral lesion OR premalignant OR manifestation OR diagnos* OR detect*) AND (dentist OR dental OR oral)

Pubmed: ("telemedicine"[MeSH Terms] OR "telemedicine"[All Fields] OR "telemedicine s"[All Fields] OR "teledentistry"[All Fields] OR ("telehealth s"[All Fields] OR "telemedicine"[MeSH Terms] OR "telemedicine"[All Fields] OR "telehealth"[All Fields]) OR "telediagnosis"[All Fields] OR "telecons*"[All Fields] OR ("telemedicine"[MeSH Terms] OR "telemedicine"[All Fields] OR "ehealth"[All Fields]) OR ("smartphone"[MeSH Terms] OR "smartphone"[All Fields] OR "smartphones"[All Fields] OR "smartphone s"[All Fields]) OR ("phone s"[All Fields] OR "phoned"[All Fields] OR "phones"[All Fields] OR "phoning"[All Fields] OR "telephone"[MeSH Terms] OR "telephone"[All Fields] OR "phone"[All Fields]) OR ("remote"[All Fields] OR "remotely"[All Fields] OR "remoteness"[All Fields] OR "remotes"[All Fields]) OR ("australas plant pathol"[Journal] OR "app"[All Fields])) AND ((("mouth"[MeSH Terms] OR "mouth"[All Fields] OR "oral"[All Fields]) AND ("lesion"[All Fields] OR "lesion s"[All Fields] OR "lesional"[All Fields] OR "lesions"[All Fields])) OR ("precancerous conditions"[MeSH Terms] OR ("precancerous"[All Fields] AND "conditions"[All Fields]) OR "precancerous conditions"[All Fields] OR "premalignant"[All Fields] OR "premalign"[All Fields] OR "premalignancies"[All Fields] OR "premalignancy"[All Fields]) OR ("manifest"[All Fields] OR "manifestating"[All Fields] OR "manifestation"[All Fields] OR "manifestations"[All Fields] OR "manifested"[All Fields] OR "manifesting"[All Fields] OR "manifestion"[All Fields] OR "manifestions"[All Fields] OR "manifests"[All Fields]) OR "diagnos*"[All Fields] OR "detect*"[All Fields]) AND ("dentist s"[All Fields] OR "dentists"[MeSH Terms] OR "dentists"[All Fields] OR "dentist"[All Fields] OR ("dental health services"[MeSH Terms] OR ("dental"[All Fields] AND "health"[All Fields] AND "services"[All Fields]) OR "dental health services"[All Fields] OR "dental"[All Fields] OR "dentally"[All Fields] OR "dentals"[All Fields]) OR ("mouth"[MeSH Terms] OR "mouth"[All Fields] OR "oral"[All Fields]))

Embase: ('telemedicine'/exp OR telemedicine OR 'teledentistry'/exp OR teledentistry OR 'telehealth'/exp OR telehealth OR 'telediagnosis'/exp OR telediagnosis OR telecons* OR 'ehealth'/exp OR ehealth OR 'smartphone'/exp OR smartphone OR phone OR remote OR app) AND ('oral lesion'/exp OR 'oral lesion' OR 'oral potentially malignant disorder'/exp OR 'oral potentially malignant disorder' OR manifestation OR diagnos* OR detect*) AND ('dentist'/exp OR dentist OR 'dental'/exp OR dental OR oral)

Cochrane: (telemedicine OR teledentistry OR telehealth OR telediagnosis OR telecons* OR ehealth OR smartphone OR phone OR remote OR app) AND (oral lesion OR premalignant OR manifestation OR diagnos* OR detect*) AND (dentist OR dental OR oral)
