# Supplementary material for: Teledentistry: A Future Solution in the Diagnosis of Oral Lesions: Diagnostic Meta-Analysis and Systematic Review
Source: Telemed J E Health. 2023 Nov 10;29(11):1591–600. doi: 10.1089/tmj.2022.0426 (PMC10654653; doi:10.1089/tmj.2022.0426)
Supplement: Supplemental data [file Suppl_DataS2.docx]

**Supplementary Material 2** Detailed Synthesis Methods

The bivariate model of Chu et al and Reitsma et al was fitted ^18, 19^ to analyze OPMD or OC detection. This approach considers the dependency between sensitivity and specificity. We plotted on an ROC plot the sensitivities and specificities of the included studies, their summary estimates, and the corresponding 95% confidence and prediction regions. In these visualizations, the sizes of the ellipsoids reflect the weights of the studies calculated according to the method described by Burke et al ^20^. In the case of oral lesion detection, when only three studies were available, separate univariate analyses of sensitivity and specificity were performed using the generalized mixed-effect approach ^21^.

Besides the prediction region, heterogeneity was assessed by calculating I² measure and its confidence interval arising from the separate univariate analyses. Statistical analyses were carried out using the online tool described in ^22^ and package meta of R statistical software (version 4.1.2.). The statistical analyses followed the advice of Harrer et al ^23^.
