# Supplementary material for: Teledentistry: A Future Solution in the Diagnosis of Oral Lesions: Diagnostic Meta-Analysis and Systematic Review
Source: Telemed J E Health. 2023 Nov 10;29(11):1591–600. doi: 10.1089/tmj.2022.0426 (PMC10654653; doi:10.1089/tmj.2022.0426)
Supplement: Supplemental data [file Suppl_TableS1.docx]

**Supplementary Table 1.** True positive, true negative, false positive, false negative values of oral lesion detection with teledentistry tools

| Author | Year | TP | FN | FP | TN |
| --- | --- | --- | --- | --- | --- |
| Haron | 2016 | 7 | 3 | 0 | 6 |
| Haron | 2021 | 254 | 22 | 25 | 52 |
| Birur | 2019 | 376 | 15 | 68 | 2955 |
