# Supplementary material for: Teledentistry: A Future Solution in the Diagnosis of Oral Lesions: Diagnostic Meta-Analysis and Systematic Review
Source: Telemed J E Health. 2023 Nov 10;29(11):1591–600. doi: 10.1089/tmj.2022.0426 (PMC10654653; doi:10.1089/tmj.2022.0426)
Supplement: Supplemental data [file Suppl_TableS2.docx]

**Supplementary Table 2.** Lesion type classification

| Group I: Cancer/ Oral Potentially Malignant Disorder (OPMD) | |
| --- | --- |
| Oral potentially malignant disease |  |
|  | Non-healing ulceration |
|  | Erythroplakia |
|  | Erythroleukoplakia |
|  | Leukoplakia (homogenous/non-homogenous) |
|  | Oral submucous fibrosis |
|  | Smokeless tobacco keratosis |
|  | Palatal lesions associated with reverse smoking |
|  | Discoid lupus erythematous |
|  | Actinic keratosis (lip only)-cheilitis actinica |
|  | Condensing ostitis |
| Malignant lesions |  |
|  | Suspicious of oral cancer |
|  | Oral squamosus cell carcinoma |
|  | Solitary pigmented macule (on palate and gingiva) |
| Group II: Benign Lesions | |
| Benign conditions of the tongue |  |
|  | Erythema migrans/ Geographic tongue |
|  | Median rhomboid glossitis |
|  | Fissured tongue |
|  | Black hairy tongue |
|  | Fimbriated fold of tongue |
| Inflammatory |  |
|  | Herpes labialis |
|  | Oral Candidiasis |
|  | Paracoccidioidomycosis |
|  | Aphthous stomatitis |
|  | Osteomyelitis |
|  | Osteoradionecrosis |
|  | Sialolithiasis |
| Benign tumours/tumour-like lesions |  |
|  | Lipoma |
|  | Pyogenic granuloma |
|  | Drug-induced gingival fibromatosis |
|  | Peripherial giant cell granuloma |
|  | Fibroma |
|  | Haemangioma |
|  | Papilloma |
|  | Mucocele |
|  | Ranula |
|  | Fibrous hyperplasia (inflammatory) |
|  | Papillary hyperplasia (inflammatory) |
|  | Necrotizing sialometaplasia |
| Other mucosal lesions |  |
|  | Denture stomatitis |
|  | Traumatic ulcer |
|  | Neutropenic ulcer |
|  | Hyperkeratosis (frictional keratosis) |
|  | Smoker’s palate |
|  | Periodontal abscess |
|  | Residual cyst |
|  | Retentional cyst |
|  | Amalgam tattoo |
|  | Neurofibromatosis |
|  | Nicotinic stomatitis |
|  | Reactive hyperkeratosis |
|  | Primary herpes stomatitis |
|  | Bone spicule |
|  | Burning mouth syndrome |
|  | Peutz-Jeghers syndrome |
| Others (related to benign/reactive) |  |
|  | Lichen planus |
|  | Erythema multiforme |
|  | Lupus erythematosus |
|  | Reconstructed tissue |
|  | Surgical defect for areas that are not reconstructed |
| Group III: Normal anatomic variant/developmental abnormalities | |
| Normal anatomic variant |  |
|  | Linea alba |
|  | Leukodema |
|  | Racial pigmentation/melanosis |
|  | Anatomical gingival fibromatosis |
|  | Diffuse pigmentation of gingiva and other parts |
|  | Varicosities |
| Developmental anomalies |  |
|  | Fordyce granules |
|  | Congenital lip pits |
|  | Palatal/mandibular torus |
|  | Ankyloglossia/tongue tie |
|  | Cleft lip/cleft palate |
|  | Exostosis |
|  | Sublingual gland eminence |
|  | Naevus |
