# Supplementary material for: Teledentistry: A Future Solution in the Diagnosis of Oral Lesions: Diagnostic Meta-Analysis and Systematic Review
Source: Telemed J E Health. 2023 Nov 10;29(11):1591–600. doi: 10.1089/tmj.2022.0426 (PMC10654653; doi:10.1089/tmj.2022.0426)
Supplement: Supplemental data [file Suppl_TableS3.docx]

**Supplementary Table 3.** True positive, true negative, false positive, false negative values of differential diagnosis of lesions

| Author | Year | TP | FN | FP | TN |
| --- | --- | --- | --- | --- | --- |
| Haron | 2016 | 7 | 1 | 0 | 8 |
| Perdoncini | 2021 | 4 | 1 | 0 | 36 |
| Torres-Pereira | 2013 | 10 | 3 | 0 | 47 |
| Haron | 2021 | 50 | 2 | 8 | 220 |
| Petruzzi | 2016 | 5 | 0 | 3 | 76 |
| Tesfaul | 2016 | 6 | 0 | 0 | 17 |
| Torres-Pereira | 2008 | 4 | 0 | 0 | 21 |
| Flores | 2022 | 20 | 0 | 1 | 79 |
| Gomes | 2017 | 14 | 3 | 0 | 38 |
