# Supplementary material for: Teledentistry: A Future Solution in the Diagnosis of Oral Lesions: Diagnostic Meta-Analysis and Systematic Review
Source: Telemed J E Health. 2023 Nov 10;29(11):1591–600. doi: 10.1089/tmj.2022.0426 (PMC10654653; doi:10.1089/tmj.2022.0426)
Supplement: Supplemental data [file Suppl_TableS4.docx]

**Supplementary Table 4****.** True positive, true negative, false positive, false negative values of differential diagnosis of lesions used in the sensitivity running. The differences compared to the input of the original running are indicated in bold.

| Author | Year | TP | FN | FP | TN |
| --- | --- | --- | --- | --- | --- |
| Haron | 2016 | 8 | 0 | 0 | 8 |
| Perdoncini | 2021 | 4 | 1 | 0 | 36 |
| Torres-Pereira | 2013 | 10 | 3 | 0 | 47 |
| Haron | 2021 | 50 | 2 | 8 | 220 |
| Petruzzi | 2016 | 5 | 0 | 3 | 76 |
| Tesfaul | 2016 | 6 | 0 | 0 | 17 |
| Torres-Pereira | 2008 | 3 | 1 | 0 | 21 |
| Flores | 2022 | 20 | 0 | 1 | 79 |
| gomes | 2017 | 17 | 0 | 7 | 31 |
