# Supplementary material for: Teledentistry: A Future Solution in the Diagnosis of Oral Lesions: Diagnostic Meta-Analysis and Systematic Review
Source: Telemed J E Health. 2023 Nov 10;29(11):1591–600. doi: 10.1089/tmj.2022.0426 (PMC10654653; doi:10.1089/tmj.2022.0426)

**Supplementary Figure 3.** Sensitivity running for diagnosing oral premalignant/malignant lesions with telendentistry tools-2D analysis


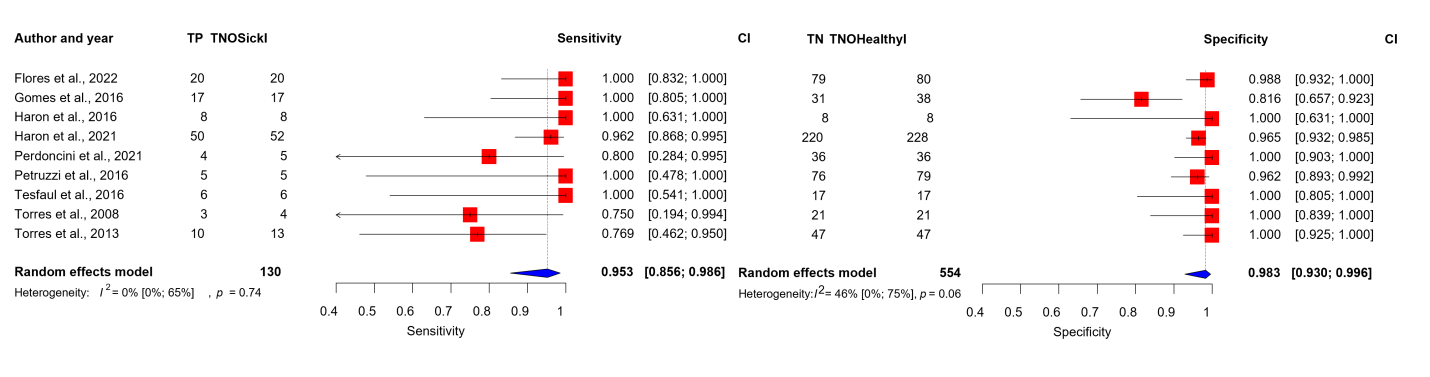

Supplement: Supplemental data [file Suppl_FigS3.docx]
