# Supplementary figures and images for: Teledentistry: A Future Solution in the Diagnosis of Oral Lesions: Diagnostic Meta-Analysis and Systematic Review
Source: Telemed J E Health. 2023 Nov 10;29(11):1591–600. doi: 10.1089/tmj.2022.0426 (PMC10654653; doi:10.1089/tmj.2022.0426)

**Supplementary Figure 4.** Risk of bias assessment for presence of oral lesion using QUADAS-2 tool


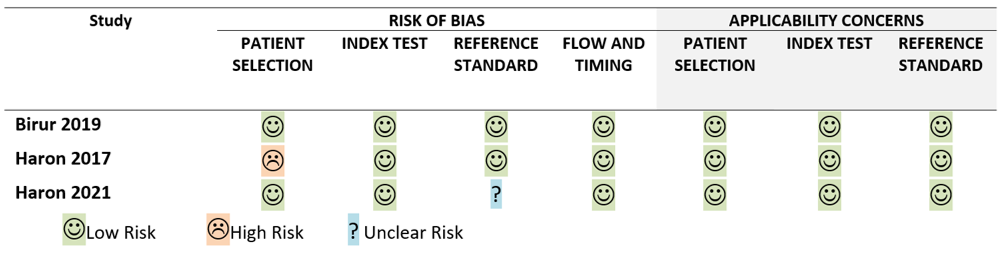

Supplement: Supplemental data [file Suppl_FigS4.docx]

**Supplementary Figure 5.** Risk of bias assessment for exact lesion diagnosis using QUADAS-2 tool


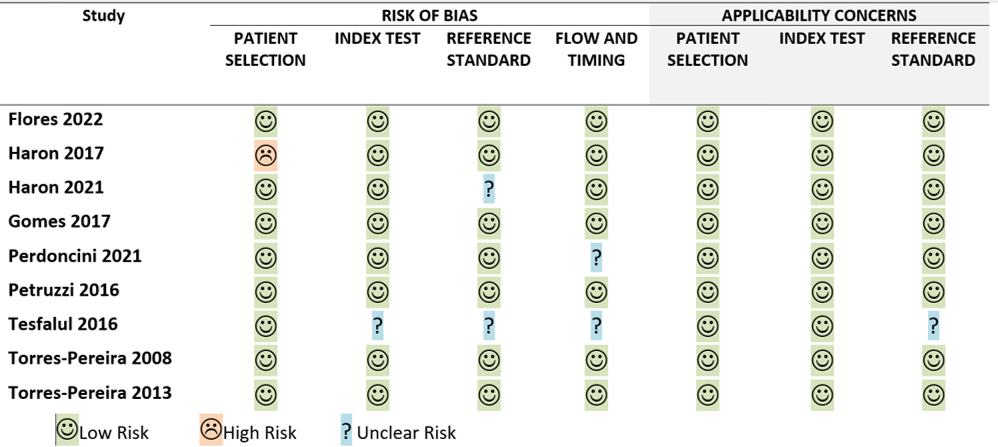

Supplement: Supplemental data [file Suppl_FigS5.docx]
