# Supplementary material for: Teledentistry: A Future Solution in the Diagnosis of Oral Lesions: Diagnostic Meta-Analysis and Systematic Review
Source: Telemed J E Health. 2023 Nov 10;29(11):1591–600. doi: 10.1089/tmj.2022.0426 (PMC10654653; doi:10.1089/tmj.2022.0426)
Supplement: Supplemental data [file Suppl_TableS5.docx]

**Supplementary Table 5.** Summary of evidence table **(**GRADE approach)

| Outcome | № of studies (№ of patients) | Study design | Factors that may decrease certainty of evidence | | | | | Effect per 100 patients tested | Test accuracy CoE |
| --- | --- | --- | --- | --- | --- | --- | --- | --- | --- |
|  |  |  | Risk of bias | Indirectness | Inconsistency | Imprecision | Publication bias | pre-test probability of 10% |  |
| True positives | 8 studies 110 patients | cross-sectional (cohort type accuracy study) | not serious | not serious | serious | not serious | publication bias strongly suspected | 9 (6 to 10) | ⨁⨁◯◯ Low |
| (patients with oral premalignant/malignant lesion) |  |  |  |  |  |  |  | 1 (0 to 4) |  |
| 110 patients | 8 studies 474 patients | cross-sectional (cohort type accuracy study) | not serious | not serious | serious | not serious | publication bias strongly suspected | 84 (15 to 90) | ⨁⨁◯◯ Low |
| Low |  |  |  |  |  |  |  | 6 (0 to 75) |  |

| Outcome | № of studies (№ of patients) | Study design | Factors that may decrease certainty of evidence | | | | | Effect per 100 patients tested | Test accuracy CoE |
| --- | --- | --- | --- | --- | --- | --- | --- | --- | --- |
|  |  |  | Risk of bias | Indirectness | Inconsistency | Imprecision | Publication bias | pre-test probability of 17.12% |  |
| **True positives** (patients with oral premalignant/malignant lesion) | 8 studies 110 patients | cross-sectional (cohort type accuracy study) | not serious | not serious | not serious | not serious | publication bias strongly suspected | 15 (14 to 16) | ⨁⨁⨁◯ Moderate |
| **False negatives** (patients incorrectly classified as not having oral premalignant/malignant lesion) |  |  |  |  |  |  |  | 2 (1 to 3) |  |
| **True negatives** (patients without oral premalignant/malignant lesion) | 8 studies 474 patients | cross-sectional (cohort type accuracy study) | not serious | not serious | not serious | not serious | publication bias strongly suspected | 82 (77 to 83) | ⨁⨁⨁◯ Moderate |
| **False positives** (patients incorrectly classified as having oral premalignant/malignant lesion) |  |  |  |  |  |  |  | 1 (0 to 6) |  |
